# Defects of the Glycinergic Synapse in Zebrafish

**DOI:** 10.3389/fnmol.2016.00050

**Published:** 2016-06-29

**Authors:** Kazutoyo Ogino, Hiromi Hirata

**Affiliations:** Department of Chemistry and Biological Science, College of Science and Engineering, Aoyama Gakuin UniversitySagamihara, Japan

**Keywords:** startle disease, glycine, receptor, synapse, transporter, zebrafish

## Abstract

Glycine mediates fast inhibitory synaptic transmission. Physiological importance of the glycinergic synapse is well established in the brainstem and the spinal cord. In humans, the loss of glycinergic function in the spinal cord and brainstem leads to hyperekplexia, which is characterized by an excess startle reflex to sudden acoustic or tactile stimulation. In addition, glycinergic synapses in this region are also involved in the regulation of respiration and locomotion, and in the nociceptive processing. The importance of the glycinergic synapse is conserved across vertebrate species. A teleost fish, the zebrafish, offers several advantages as a vertebrate model for research of glycinergic synapse. Mutagenesis screens in zebrafish have isolated two motor defective mutants that have pathogenic mutations in glycinergic synaptic transmission: *bandoneon* (*beo*) and *shocked* (*sho*). *Beo* mutants have a loss-of-function mutation of glycine receptor (GlyR) β-subunit b, alternatively, *sho* mutant is a glycinergic transporter 1 (GlyT1) defective mutant. These mutants are useful animal models for understanding of glycinergic synaptic transmission and for identification of novel therapeutic agents for human diseases arising from defect in glycinergic transmission, such as hyperekplexia or glycine encephalopathy. Recent advances in techniques for genome editing and for imaging and manipulating of a molecule or a physiological process make zebrafish more attractive model. In this review, we describe the glycinergic defective zebrafish mutants and the technical advances in both forward and reverse genetic approaches as well as *in vivo* visualization and manipulation approaches for the study of the glycinergic synapse in zebrafish.

## Overview of Glycinergic Neurotransmission in the Mammalian Nervous System

Glycine is a major inhibitory neurotransmitter in the brainstem and spinal cord. The glycinergic transmission plays important roles in the reflex and the control of rhythmic motor behaviors such as locomotion and breathing (Schmid et al., 1991, 1996; Gomeza et al., 2003a; Grillner, 2003; Callister and Graham, 2010), as well as nociceptive processing (Baba et al., 2001; Ahmadi et al., 2002; Harvey et al., 2004; Zeilhofer, 2005). Glycine receptors (GlyRs) are also expressed in the retina (Sassoè-Pognetto et al., 1994; Haverkamp et al., 2003, 2004; Jusuf et al., 2005; Heinze et al., 2007) and various regions of the brain, such as amygdala (McCool and Botting, 2000; McCool and Farroni, 2001), hippocampus (Malosio et al., 1991; Chattipakorn and McMahon, 2002, 2003; Brackmann et al., 2004; Danglot et al., 2004; Eichler et al., 2009; Jonsson et al., 2012; Chen et al., 2014; Winkelmann et al., 2014; Çalişkan et al., 2016), midbrain (Malosio et al., 1991; Mangin et al., 2002; Jonsson et al., 2012), neocortex (Malosio et al., 1991; Jonsson et al., 2012; Salling and Harrison, 2014), to regulate neuronal network excitability. GlyRs are pentameric ligand-gated chloride channels taking the form of α-subunit homopentamers or αβ-subunit heteropentamars. The α-subunit is ligand-binding subunit (Becker et al., 1988; Grenningloh et al., 1990a,b; Kuhse et al., 1990), whereas the β-subunit binds to synaptic gephyrin scaffold consisting of tens to hundreds gephyrin molecules, thereby the αβ heteromeric GlyRs can be accumulated at postsynaptic sites (Kirsch et al., 1993; Meyer et al., 1995; Feng et al., 1998; Hanus et al., 2004; Sola et al., 2004; Grudzinska et al., 2005; Kim et al., 2006; Calamai et al., 2009; Herweg and Schwarz, 2012; Specht et al., 2013). However, the postsynaptic localization of GlyR is not permanent. GlyRs are constantly exchanged between synaptic sites and extrasynaptic plasma-membrane by means of lateral diffusion, and alterations of the diffusion properties have been considered to be mechanisms involved in the synaptic plasticity (Meier et al., 2001; Dahan et al., 2003; Ehrensperger et al., 2007; Lévi et al., 2008; Calamai et al., 2009; Specht et al., 2011). Furthermore, the postsynaptic gephyrin scaffold is involved in regulation of the properties for synaptic plasticity in both glycinergic synapses and GABAergic synapses through interactions with various proteins (Fritschy et al., 2008; Tyagarajan and Fritschy, 2014).

Two possible stoichiometric models of the heteromeric GlyR have been proposed from research using purified GlyR subunits: 3α2β and 2α3β (Langosch et al., 1988; Burzomato et al., 2003; Grudzinska et al., 2005). Durisic et al. (2012) published data supporting 3α2β stoichiometry by single-molecule stepwise photo-bleaching: whereas a homomeric channel of fluorescent protein-tagged α subunits exhibited bleaching by a five-step reduction of fluorescence, a heteromeric channel containing the fluorescent protein-tagged α-subunits exhibited bleaching by a three-step reduction (Durisic et al., 2012). However, another group proposed the likelihood of the 2α3β stoichiometry based on the observation of antibody bounded receptor consisting of FLAG-tagged α-subunit and His-tagged β-subunit using single-molecule resolution atomic force microscopy (Yang et al., 2012). The single-molecular imaging showed that the maximum binding number of antibodies to each of the tag, anti-FLAG antibodies and anti-His antibodies could bind to the receptor up to two and three, respectively. The exact stoichiometry of the αβ GlyR heteromer thus remains a point of contention, and requires further investigation. Although the formation of a homomeric β GlyR has also been hypothesized, biochemical evidence has indicated that β subunits do not form pentamers (Griffon et al., 1999), and an electrophysiological analysis showed that recombinant expression of only the β subunit in HEK-293 cells does not result in glycine-gated currents (Bormann et al., 1993).

In contrast to heteromeric GlyR, homomeric GlyRs are mainly distributed in the extrasynaptic area or presynaptic terminal in the brain and spinal cord (Turecek and Trussell, 2001; Jeong et al., 2003; Ye et al., 2004; Wang et al., 2005; Deleuze et al., 2005; Eichler et al., 2008; Kubota et al., 2010; Hruskova et al., 2012; Kunz et al., 2012; Avila et al., 2013; Chen et al., 2014; Salling and Harrison, 2014; Winkelmann et al., 2014). The extrasynaptic homomeric GlyRs are activated by ambient glycine or taurine, thereby tonic glycinergic current that contributed to regulation of neuronal excitability both in mature and immature neural tissue is evoked (Chattipakorn and McMahon, 2003; Wang et al., 2005; Eichler et al., 2008; Chen et al., 2014; Maguire et al., 2014; Salling and Harrison, 2014). In contrast to the GlyRs on mature neurons, GlyRs on immature neurons typically induce depolarizing chloride efflux in accordance with the chloride gradient that is opposite to that of mature neurons (Reichling et al., 1994; Flint et al., 1998; Ben-Ari, 2002). The inverted chloride gradient is formed and maintained by Na^+^-K^+^-2Cl^-^ cotransporter (NKCC1), which import chloride ions into the cell. On immature neurons, presynaptic GlyRs facilitate neurotransmitter release through activation of voltage gated calcium channel (VGCC) (Turecek and Trussell, 2001; Jeong et al., 2003; Ye et al., 2004; Eichler et al., 2009; Kunz et al., 2012; Winkelmann et al., 2014). Similarly, tonic activation of the extrasynaptic GlyR on the developing immature neurons induced Ca^2+^ transients through VGCCs. The activation of VGCCs have been considered to promote the synaptic accumulation of GlyRs (Kirsch and Betz, 1998; Kneussel and Betz, 2000) or to regulate the development of cortical neurons by promoting migration (Avila et al., 2013, 2014). The Ca^2+^ transients may also be involved in the K^+^-Cl^-^ cotransporter type 2 (KCC2) expression (Brustein et al., 2013; Allain et al., 2015). It is generally accepted, the activity of NKCC1 declines with progress of development, whereas activity of KCC2, which establishes the chloride gradient of mature neurons by extrusion of chloride ions, becomes predominant (Kanaka et al., 2001; Ben-Ari, 2002; Mikawa et al., 2002; Wang et al., 2002; Delpy et al., 2008). The KCC2 activity is necessary for glycinergic synapse maturation. Knockdown of KCC2 impaired cluster formation of GlyR α1 subunit (adult-type subunit) and gephyrin in cultured spinal neurons, whereas α2 subunit (neonatal type subunit) cluster formation was not affected by the KCC2 knockdown (Schwale et al., 2016). In addition to the developmental role, the tonic depolarizing current contributes to regulate the neuronal excitability in immature hippocampus (Chattipakorn and McMahon, 2003; Eichler et al., 2008; Chen et al., 2014).

The α homomeric GlyR current can be pharmacologically distinguished from that of the αβ heteromeric GlyR by picrotoxin, a GlyR inhibitor: picrotoxin has a 100-fold greater affinity for the α homomeric GlyR than the αβ heteromeric GlyR, such that the α homomeric GlyR is specifically blocked by picrotoxin at low concentrations (Pribilla et al., 1992; Mangin et al., 2002). These currents can also be distinguished by electrophysiological analysis, because the conductance mediated by α1 or α3 homomeric GlyR is larger than that of α1β or α3β heteromeric GlyR (Takahashi et al., 1992; Bormann et al., 1993; Rajendra et al., 1995; Beato et al., 2002; Burzomato et al., 2004; Zhang et al., 2015).

In addition to the GlyR agonist, glycine acts as co-agonist of *N*-methyl-D-aspartate receptor (NMDAR) (Johnson and Ascher, 1987). The simultaneous binding of glutamate and the co-agonist is required to activate the NMDAR (Johnson and Ascher, 1987; Furukawa et al., 2005; Paoletti and Neyton, 2007). Although the physiological significance of co-agonist glycine is unclear, the recent works have reported the involvement in induction of LTD (Papouin et al., 2012), morphogenesis of dopaminergic neurons (Schmitz et al., 2009, 2013).

### Mammalian Glycine Receptor Subunits

There exist four GlyR α subunit genes (*GLRA1, GLRA2, GLRA3, GLRA4*) and one β subunit gene (*GLRB*) in humans and rodents (Grenningloh et al., 1987, 1990b; Kuhse et al., 1990, 1991; Harvey et al., 2000), although the human *GLRA4* is not expressed due to a premature stop codon in this gene, thus human *GLRA4* is pseudogene (Simon et al., 2004). Electrophysiological studies on cultured cells expressing mammalian GlyR subunits have shown that the difference in conductance and kinetics between α1β/α3β and α2β, the conductance of α2β is larger than that of the former, activation kinetics of α2 homomeric and α2β heteromeric is slower than α1/α3 containing receptor (Takahashi et al., 1992; Bormann et al., 1993; Rajendra et al., 1995; Beato et al., 2002; Mangin et al., 2003; Burzomato et al., 2004; Zhang et al., 2015). The adult mammalian hindbrain and spinal cord predominantly expresses the α1 (*GLRA1*) and β (*GLRB*) GlyR subunits (Malosio et al., 1991; Singer et al., 1998; Jonsson et al., 2012; Weltzien et al., 2012). Hence, it is assumed that heteromeric α1β GlyRs mediate a majority of glycinergic neurotransmission in these matured neural tissues. In this physiological context, it has been shown that glycinergic synaptic transmission is important for the generation of rhythmic motor behaviors. Functional loss of human *GLRA1* due to missense, nonsense, or frame-shift mutation leads to the development of a hyperekplexia syndrome that is characterized by various exaggerated startle responses to unexpected acoustic or tactile stimuli, as well as neonatal apnea (Harvey et al., 2008a; Davies et al., 2010; Bode and Lynch, 2014). In addition, mutations that are associated with hyperekplexia syndrome have been identified in the GlyR β subunit gene (Rees et al., 2002; Al-Owain et al., 2012; Chung et al., 2013; James et al., 2013; Mine et al., 2013; Rizk and Mahmoud, 2014), the gephyrin gene (Rees et al., 2003), and the collybistin gene (Harvey et al., 2004). Furthermore, mutation of the glycine transporter 2 (GlyT2) gene (*slc5a6*) leads to a reduction in presynaptic glycine release and also produces hyperekplexia (Harvey et al., 2008a).

Also α3 subunits are expressed in the spinal cord as heteromeric GlyRs. The α3 subunit distribution is restricted to the superficial layer of the adult dorsal horn of the spinal cord (Harvey et al., 2004), and the α3 subunit is involved in nociceptive processing by modulating the excitability of projection neurons that relay nociceptive input from peripheral afferents (Harvey et al., 2004, 2009; Zeilhofer, 2005). Prostaglandin E2, an important inflammatory mediator, induces protein kinase A-dependent phosphorylation at Ser346 of the α3 subunit to depress glycinergic synaptic transmission and facilitate inflammatory pain (Ahmadi et al., 2002; Harvey et al., 2004; Zeilhofer, 2005). Although the α3 knockout mice exhibited neither morphological abnormality nor neuro-motor phenotype, the prostaglandin E2 induced pain sensitization was abolished in the α3 knockout mice (Harvey et al., 2004). Hence the α3 subunit may therefore be a promising target for the regulation of inflammatory pain. In fact, cannabinoids that potentiate α3 subunit exhibited analgesic effect to chronic inflammatory pain without psychoactive side effect (Xiong et al., 2012). The α3 phosphorylation induced conformational change specifically on glycine binding site of the α3 subunit, because the serine residues is not conserved at the equivalent position of the α1 subunit (Harvey et al., 2004; Han et al., 2013). The specific conformational change may be a help to develop other type specific drugs potentiating the phosphorylated α3 subunit. Thus, the glycinergic synaptic transmission in spinal cord is mediated by the α1β and α3β heteromeric receptor, and synaptic transmission is an attractive for the development effective remedies. In addition to the pain regulation in spinal cord, changes in the activity of hippocampal α3 subunit are also associated with temporal lobe epilepsy (Eichler et al., 2008; Legendre et al., 2009; Winkelmann et al., 2014).

The α2 subunit mRNA encoded by *GLRA2* gene is predominantly expressed in the developing spinal cord, and then the α2 subunit is largely replaced by the α1 subunit in this regions within 2 weeks after birth in mice (Kuhse et al., 1990; Malosio et al., 1991; Watanabe and Akagi, 1995; Singer et al., 1998; Liu and Wong-Riley, 2013). Functional α2 homomeric GlyRs also found in embryonic immature cortex neurons (Flint et al., 1998; Young-Pearse et al., 2006). Although a previous study using α2 knockout mice showed no morphological or molecular alterations in nervous system development (Young-Pearse et al., 2006), recent analysis in newly established α2 knockout mice indicated that the α2 subunit contributes to several neural development process, such as tangential migration in developing cortex (Avila et al., 2013), cerebral cortical neurogenesis (Avila et al., 2014), morphogenesis and synaptogenesis of somatosensory cortical neuron (Morelli et al., 2016). The importance of α2 subunit in development and maturation of brain was also underscored by the recent identification of a micro-deletion and two mutations in GLRA2 gene from patients with autism spectrum disorder (Pinto et al., 2010; Pilorge et al., 2015).

After the developmental switching in the spinal cord, α2 and α3 subunits are still expressed as the predominant subunits in some regions of adult brain such as hippocampus and frontal cortex; in these regions the GlyRs contribute to regulation of neural excitability and synaptic plasticity (Chattipakorn and McMahon, 2002; Song et al., 2006; Zhang et al., 2006, 2008; Eichler et al., 2009; Kubota et al., 2010; Aroeira et al., 2011; Jonsson et al., 2012). GlyR β subunit mRNA was abundantly detected throughout the embryonic and adult brain, from olfactory bulb to spinal cord (Fujita et al., 1991; Malosio et al., 1991). However, a recent immunohistochemical study using a novel monoclonal antibody to the GlyR β subunit exhibited distinctive punctate staining of the β subunit at synaptic sites only in spinal cord, brainstem, midbrain, olfactory bulb, and retina of adult mice (Weltzien et al., 2012). In contrast to these regions, only weak diffuse immunostaining signals were detected in the hypothalamus, the cerebellum, the hippocampus and the neocortex of adult mice (Weltzien et al., 2012). These observations suggest that most of GlyRs in adult brain are extrasynaptic homopentamer, as presented in previous studies about hippocampal GlyR (Chattipakorn and McMahon, 2002; Zhang et al., 2008; Aroeira et al., 2011). It has been suggested that the tonic inhibition by the neocortex GlyRs have antiepileptic effect (Chattipakorn and McMahon, 2003; Kirchner et al., 2003; Zhang et al., 2008; Shen et al., 2015). Epilepsy upregulated the expression of high-affinity GlyR through RNA editing in GlyR mRNA; this high-affinity GlyR may facilitate tonic inhibition to suppress the epileptic activity (Eichler et al., 2008).

In other region of adult brain, GlyRs are considered to participate in drug addiction. Studies using rodent models have revealed that some of the cortex GlyRs are involved in the behavioral effect of ethanol, such as a motor incoordination or a hypnosis (Quinlan et al., 2002; Ye et al., 2009; Blednov et al., 2012; McCracken et al., 2013a,b; Aguayo et al., 2014), and in the ethanol preference (Blednov et al., 2015). Ethanol increases extracellular dopamine levels in nucleus accumbens of the brain reward system through enhancement of the accumbal GlyR activity (Molander and Söderpalm, 2005; Molander et al., 2005; Maguire et al., 2014). The accumbal GlyRs are also involved in the dopamine-elevating effects of tetrahydrocannabinol and nicotine in the nucleus accumbens (Jonsson et al., 2014). Since dopamine elevation is a basis of drug addiction, the accumbal GlyR could be therapeutic targets of the addiction of ethanol or nicotine.

In addition to the expression within the brain, immunolabeling studies have demonstrated that all α subunits are also expressed in retina, especially in inner plexiform layer (IPL), with subunit -specific expression patterns (Sassoè-Pognetto et al., 1994; Haverkamp et al., 2003, 2004; Jusuf et al., 2005; Heinze et al., 2007; Weiss et al., 2008; Zhang et al., 2014), suggesting that different GlyR α subunits may be involved in different levels of visual processing (Wässle et al., 2009). Monoclonal antibody against the GlyR β subunit revealed that the β subunits were almost completely colocalized with GlyR α1-3 subunits and gephyrin in the IPL of mouse retina, however, GlyR α4 subunit did not show the highly colocalization with the β subunits (Weltzien et al., 2012). Thus, most of the retinal GlyR are αβ heteromeric receptor. The physiological role of these glycinergic synapses have been investigated by pharmacological and genetic approaches. For example, strychnine, a specific GlyR blocker, mediated blockade of glycinergic feedback pathway that from neuron in IPL to photoreceptor cell reduced the amplitudes of light-evoked response in both ON and OFF bipolar cells, indicating that the feedback pathway regulates signal propagation in the distal retina (Jiang et al., 2014). Genetic manipulations of GlyR α subunits *in vivo* have informed the significance of GlyR α subunit expression in the retina. Inhibitory glycinergic transmission between amacrine and bipolar cells of the retina is absent in *GLRA1*-deficient mice (Ivanova et al., 2006), and the frequency of spontaneous glycinergic input to A-type ganglion cells is also significantly reduced in the *GLRA1*-deficient retina (Majumdar et al., 2007). A study of *GLRA2* knockout mice revealed that α2-containing GlyRs are involved in glycinergic input to group II wide-field amacrine cells (Majumdar et al., 2009). In the *GLRA2*-deficient retina, glycine-evoked currents are also reduced in MA-S5 and ON-starburst amacrine cells, suggesting that the α2 subunit is also involved in information processing by the amacrine cells. In *GLRA3* deficient mice, spontaneous glycinergic currents are absent in AII amacrine cells (Weiss et al., 2008), additionally suggesting a role for the α3 subunit in these cells. In addition, α2 and α3 subunits enhance the excitatory center response of retinal receptive fields through modulation of local receptive field interactions (Nobles et al., 2012). Since the kinetics of glycinergic transmission differ according to α subunit type, signaling via different GlyRs may facilitate the complex regulation of retinal neuronal networks in visual processing.

### Mammalian Glycine Transporters

Two glycine transporters have been identified in the mammalian CNS: glycine transporter 1 (GlyT1) and 2 (GlyT2). Both of these transporters belong to the Na^+^/Cl^-^ dependent transporter family and mediate the uptake of glycine from the extracellular space into the cytosol (Eulenburg et al., 2005; Betz et al., 2006). GlyT1 is primarily expressed in glial cells throughout the CNS (Eulenburg et al., 2005; Betz et al., 2006). Glycinergic transporter 1 regulates glycine concentrations in the synaptic cleft of glycinergic synapses and glutamatergic synapses, where glycine acts as an essential co-agonist of NMDAR (Eulenburg et al., 2005; Betz et al., 2006). GlyT1 knockout mice display respiratory insufficiency as a result of elevated synaptic glycine and over-activation of GlyRs in the respiratory pathway (Gomeza et al., 2003a). Alternatively, GlyT2 is expressed in glycinergic neurons and mainly localizes to the presynaptic terminal. GlyT2 facilitates the uptake of glycine released into the presynaptic terminal in order to limit the extent of neurotransmission and allow the recycling of glycine for future synaptic transmission. Accordingly, GlyT2 knockout mice display attenuated glycinergic synaptic transmission (Gomeza et al., 2003b).

## Glycinergic Neurotransmission in Non-Mammalian Systems

Many investigations to reveal the physiological role of glycinergic synapse have been conducted in the mammalian model animals or human; however, non-mammalian vertebrates have been used for investigation the physiological roles of glycinergic synapse in locomotive behaviors (Drapeau et al., 2002; Grillner, 2003; Korn and Faber, 2005; Roberts et al., 2008). Most teleosts have Mauthner cells (M-cells), which are paired large neurons found in their hindbrain (Eaton et al., 1977; Zottoli, 1977). The M-cell is activated by auditory input, and the M-cell firing triggers fast escape behaviors (Kohashi and Oda, 2008; Kohashi et al., 2012). Both excitatory (e.g., glutamatergic) and inhibitory (e.g., glycinergic and GABAergic) synapses are formed on dendrites and soma of the M-cell (Korn and Faber, 2005). In goldfish, glycinergic input to M-cells was enhanced by tetanization of the auditory pathway (Korn et al., 1992) or repeated sound stimulation (Oda et al., 1998). Glycinergic synaptic transmission reduces M-cell excitability by countering concurrent excitatory synaptic input (Korn and Faber, 2005; Curtin and Preuss, 2015). Thus, the plasticity of the glycinergic synapse has been shown to regulate auditory conditioning of the escape response (Oda et al., 1998). Recently, zebrafish (*Danio rerio*) have also been used as a model for investigating the physiological roles of glycinergic neurotransmission (Cui et al., 2005; Hirata et al., 2005; Downes and Granato, 2006; Rigo and Legendre, 2006; Mongeon et al., 2008). In this review, we will describe advances in both forward and reverse genetic approaches as well as visualization techniques that have proven helpful for the study of the glycinergic synapse in zebrafish.

### Zebrafish Glycine Receptors

In total, zebrafish express five α subunits (*glra1*, *glra2*, *glra3*, *glra4a* and *glra4b*) and two β subunits (*glrba* and *glrbb*) (Hirata et al., 2010). Four GlyR α subunits (αZ1, αZ2, αZ3, and αZ4) and two β subunits (βa/βZ and and βb) were initially reported in zebrafish (David-Watine et al., 1999; Imboden et al., 2001a,b,c; Hirata et al., 2005). Although αZ2 was originally thought to encode a GlyR α2 subunit (Imboden et al., 2001a), the gene was subsequently reclassified as a second α4 subunit in a more detailed phylogenetic analysis (Imboden et al., 2001b). Thus, αZ2 was designated *glra4a*. Phylogenetic analyses have suggested that αZ1, αZ3, and αZ4 subunits are orthologs of the mammalian α1, α3, and α4 GlyR subunits, respectively (Imboden et al., 2001b); thus, these subunits have been referred to as the zebrafish GlyRα1, GlyRα3, and GlyRα4b. Of note, the existence of distinct orthologs of a mammalian gene is common in the zebrafish genome due to suspected whole genome duplication during fish evolution (Amores et al., 1998). The spatial expression of GlyR genes has been examined using *in situ* hybridization (**Table 1**). Although the spatial expression patterns of *glra2* and *glra3* remain to be determined, quantitative PCR analysis of developing zebrafish embryos (24–72 hpf) has revealed the temporal expression patterns for these genes: *glra1*, *glra3*, and *glra4a* were observed at 24 hpf, whereas *glra2* expression was not induced until 32 hpf (Ganser et al., 2013). Moreover, the expression levels of *glra1, glra2, and glra4a* were noted to steadily increase during development, while the expression of *glra3* subsided after 48 hpf (Ganser et al., 2013). Knock down experiments using antisense morpholino oligo (MO) have reported the specific involvement of *glra4a* in the differentiation of spinal interneurons (McDearmid et al., 2006).

**Table 1:** Spatial expression pattern of glycine receptor subunits

Gene	Stage	Expressing tissue	Reference
glra1	24 hpf	Telencephalon, posterior part of midbrain midbrain-hindbrain boundary reticular neurons of hindbrain, spinal neurons, eye primordium	Devignot et al., 2003 Hirata et al., 2005
	48 hpf	Spinal neurons	McDearmid et al., 2006
	52 hpf	Telencephalon, Diencephalon, Midbrain, Hindbrain, Spinal cord	Devignot et al., 2003

glra2		Remain to be determined	

glra3		Remain to be determined	

glra4a	24 hpf	Subset of cells in the telencephalon, rhombic lip, midbrain-hindbrain boundary region, reticular neurons of hindbrain, spinal neuron, somite	Imboden et al., 2001b McDearmid et al., 2006
	52 hpf	Olfactory pit, midbrain, reticular neurons of hindbrain, somite	Imboden et al., 2001b

glra4b	24 hpf	Rhombensephalic lip, midbrain hindbrain boundary region	Imboden et al., 2001b
	52 hpf	Ganglion cell layer of retina	Imboden et al., 2001b; Hensley et al., 2011

glrba		Remain to be determined	

glrbb	24 hpf	Reticular neurons of hindbrain, spinal neuron	Hirata et al., 2005

### The Advantages of Zebrafish as a Vertebrate Model

Zebrafish offer several advantages as a model for vertebrate development. First, zebrafish quickly reach sexual maturity (within 3 months), and the adult zebrafish lay 100–200 eggs twice a week, such that fertilized eggs are readily available throughout the year with a relatively low cost, because a large number of zebrafish can be maintained in smaller space compared to model mammals. Second, zebrafish embryos are optically transparent, and their development progresses rapidly at 28.5°C (Kimmel et al., 1995). These characteristics are helpful for developmental studies *in vivo*. Indeed, *in vivo* imaging of events such as synapse formation and subcellular CaMKII translocation has been achieved through the use of green fluorescent protein tagging in zebrafish embryos (Gleason et al., 2003; Niell et al., 2004). Moreover, the transparency of early zebrafish embryos has enabled the *in vivo* study and manipulation of neural activity (Douglass et al., 2008; Arrenberg et al., 2009; Zhu et al., 2009; Schoonheim et al., 2010; Muto et al., 2011). While the transparency of the zebrafish embryo is progressively diminished by the formation of melanophores after 24 hpf, 1-phenyl 2-thiourea (PTU) is a widely used agent to prevent the pigment formation (Karlsson et al., 2001). Alternatively, because it is not feasible to grow zebrafish into adulthood in the presence of PTU, a pigment-deficient mutant known as *casper* is now available, and facilitates the *in vivo* visualization and manipulation of neural activity in adult zebrafish (White et al., 2008). Third, zebrafish are suitable for electrophysiological analyses. The activity of neurons and muscles can be recorded using standard patch-clamp and extracellular recording techniques during most stages of zebrafish development (Grunwald et al., 1988; Legendre and Korn, 1994, 1995; Ribera and Nüsslein-Volhard, 1998; Drapeau et al., 1999; Saint-Amant and Drapeau, 2001; Sidi et al., 2003; Higashijima et al., 2004; Kimura et al., 2006; Fetcho, 2007; McLean et al., 2007; Tanimoto et al., 2009). Finally, the high-efficient mutagenesis and transgenesis are available in zebrafish, as discussed in a later section of this review.

In addition to the usefulness in developmental study, zebrafish is also valuable model for high-throughput screening of novel drugs (Peterson et al., 2000; Goldsmith, 2004; Love et al., 2004). The high-throughput screening using mutant zebrafish model of human disease have identified novel compound that potentially ameliorate the phenotype associated with the mutation (Peterson et al., 2004; Cao et al., 2009; Paik et al., 2010; Kawahara et al., 2011; Peal et al., 2011; Baraban et al., 2013).

### The Development of Locomotive Behavior

Zebrafish exhibit three distinct behaviors during embryogenesis: spontaneous coiling, touch-evoked escape contractions, and swimming. Spontaneous coiling appears after 17 hpf, and consists of side-to-side alternating contractions of the axial muscles (Saint-Amant and Drapeau, 1998; Downes and Granato, 2006; Pietri et al., 2009). The frequency of spontaneous coiling reaches a peak of 0.3–1 Hz at 19 hpf and gradually declines to less than 0.1 Hz by 26 hpf. After 21 hpf, zebrafish embryos respond to tactile stimuli with escape contractions that typically consists of two-to-three rapid, alternating contractions of the axial muscles (Saint-Amant and Drapeau, 1998; Hirata et al., 2005). By 28 hpf, tactile stimuli initiate swimming (Saint-Amant and Drapeau, 1998). The frequency of swimming contractions reaches 30 Hz at 36 hpf, which is comparable to the frequency of tail contraction in adult zebrafish (Buss and Drapeau, 2001). Thus, the neural network regulating swimming is functionally matured within 1.5 days of development. Interestingly, head-removed embryos transected at somites 5–7 are capable of responding to touch with an initial tail flip, although swimming fails to follow in most cases (Downes and Granato, 2006). Indeed, detailed observations suggest that the spinal cord can initiate touch responses, while supraspinal input is necessary for swimming. The spinal cord region located between somites 5–10 somite appears to be responsible for spontaneous coiling (Pietri et al., 2009). Given this knowledge, a variety of *in vivo* and *ex vivo* manipulations are possible in zebrafish.

### Mutagenesis and Transgenesis in Zebrafish

Mutagenesis for the forward genetic screening of zebrafish mutants was first established in the early 1990’s (Mullins et al., 1994), and two large-scale *N*-ethyl-*N*-nitrourea (ENU)-based mutagenesis projects were completed in Tübingen, Germany and Boston, USA by 1996. These screens identified more than 4,000 mutants, including motility defective mutants, caused by the dysfunction of glycinergic neurotransmission.

Additionally, more comprehensive ENU-based mutagenesis project named “Zebrafish Mutation Project (ZMP)” was launched in 2011. ZMP identified potentially disruptive mutations in more than 38% of all known zebrafish protein-coding genes (Kettleborough et al., 2013). The forward genetic approach provides an opportunity to identify novel genes participating in the investigating subject. The growing list of disruptive mutated alleles will be a valuable resource both for fundamental and clinical research to reveal the interested gene function.

On the other hand, reverse genetic approaches has also been applied to zebrafish biology. Targeting-induced local lesions in genomes (TILLING) has been introduced to zebrafish as the first available reverse genetics method (Wienholds et al., 2002). TILLING is a combinational method of chemical-induced mutagenesis and high-throughput screening; accordingly, TILLING can generate not only loss of function mutations, but also unexpected mutations due to non-directional missense mutagenesis (Wienholds et al., 2002, 2003; Moens et al., 2008). However, TILLING is time-consuming and less effective for intron-rich genes due to a decreased chance of obtaining null or hypomorphic alleles (Stemple, 2004). Subsequently, Zinc finger nucleases (ZFNs) (Doyon et al., 2008; Meng et al., 2008; Foley et al., 2009; Sander et al., 2011a) and transcription activator-like effector nucleases (TALEN) (Sander et al., 2011b; Bedell et al., 2012; Cade et al., 2012; Dahlem et al., 2012) were introduced to zebrafish genetics as targeted mutagenesis methods. ZFNs utilize an array of zinc finger DNA-binding motifs that bind to specific DNA triplet sequences (Urnov et al., 2005; Carroll, 2011). However, the exact DNA binding specificity of ZFNs has not been completely resolved (Carroll, 2011) and thus each ZFN construct requires careful design and screening of its target DNA-binding zinc finger motifs. Alternatively, TALEN DNA binding specificity is more predictable (Miller et al., 2011). The DNA-binding motif of TALEN is composed of repeated modules, where each module independently binds to a specific single nucleotide through repeat variable di-residues in a 1:1 manner. Thus, each module is a functional unit for the recognition of DNA sequences (Boch et al., 2009; Moscou and Bogdanove, 2009). This simple rule of nucleotide recognition has made TALEN more popular than ZFN for zebrafish genetics.

More recently, the clustered regularly interspaced palindromic repeats/CRISPR-associated 9 (CRISPER/Cas9) method was applied to zebrafish as a surprisingly simple and effective method. CRISPR and Cas are involved in bacterial adaptive immunity against the invasion of foreign nucleic acids derived from exogenous plasmids or bacteriophages (Barrangou et al., 2007; Garneau et al., 2010; Horvath and Barrangou, 2010). In type II CRISPR/Cas systems, Cas9 nuclease is guided to the target site by two RNA molecules, tracrRNA and crRNA. Alternatively, the use of a single chimeric guide RNA, generated by fusing the 3′ end of a crRNA to the 5′ end of a tracrRNA, has been used to guide Cas9 to its target site (Jinek et al., 2012). Thus, instead of the need to design a DNA binding domain consisting of several 10–100s amino acids as in the case of ZFN and TALEN, a single small guide RNA can be used in CRISPR/Cas9. Codon-optimized Cas9 with guide RNA efficiently induces target sequence-specific genome modification in humans and mammals (Cong et al., 2013; Li D. et al., 2013; Li W. et al., 2013; Mali et al., 2013). In zebrafish, CRISPR/Cas9 produces targeted gene lesion with a similar efficiency to that of TALENs (Chang et al., 2013; Hwang et al., 2013). Furthermore, the mutagenesis efficiency was improved with zebrafish codon optimized Cas9 (Jao et al., 2013; Liu et al., 2014). The off-target effects of the CRISPR/Cas9 method are reported to be quite limited (Chang et al., 2013; Hruscha et al., 2013; Hwang et al., 2013; Jao et al., 2013), although some studies in cultured cells have reported high off-target mutagenesis (Fu et al., 2013; Hsu et al., 2013). This complication can be resolved through back-crossing with wild-type zebrafish. CRISPE/Cas9 system is able to introduce not only an in-del mutation leading to gene knockout, but also exogenouse coding DNA in site specific manner (Auer et al., 2014; Kimura et al., 2014; Hisano et al., 2015). The site specific insertion allows researchers to substitute any nucleotides or amino acids, or to tagging any protein with fluorescent protein or epitope tag.

Other than the knockout technologies, MO injection into fertilized eggs has been widely used as gene knockdown method (Nasevicius and Ekker, 2000). However, in some cases MO-induced phenotypes (morphant) were not observed to correspond with equivalent mutants generated by ZFN and TALEN (Kok et al., 2015). Phenotypic discrepancies between morphants and mutants may be due to the off-target effects of MO (Kok et al., 2015). Alternatively, genetic compensation induced by deleterious mutation has also been reported as a cause of phenotype variation between morphants and mutants, as compensatory gene upregulation is not typically observed in MO-injected embryos (Rossi et al., 2015). Regardless of the cause of phenotypic discrepancy, antisense MOs should be used with meticulous care, and consider dose dependency as well as the implementation of proper positive and negative controls.

Transposon or retroviral methods have also been used to generate transgenic zebrafish (Davidson et al., 2003; Kawakami et al., 2004; Ellingsen et al., 2005; Villefranc et al., 2007; Asakawa et al., 2008) with improved efficiency (Davidson et al., 2003; Kawakami et al., 2004; Ellingsen et al., 2005; Sivasubbu et al., 2006; Villefranc et al., 2007). This increased efficiency has facilitated the use of the yeast GAL4/UAS system (Scheer and Campos-Ortega, 1999; Inbal et al., 2006; Scott et al., 2007; Asakawa et al., 2008; Halpern et al., 2008). GAL4, a yeast transcriptional activator, is capable of binding UAS sequences and driving the expression of downstream genes (Brand and Perrimon, 1993; Asakawa and Kawakami, 2008). Thus, a gene of interest can be expressed in any tissue by crossing UAS transgenic zebrafish with tissue-specific GAL4 driver transgenic zebrafish. Recently, various GAL4 driver transgenic zebrafish have been established using the Tol2 transposon-mediated enhancer trap method (Asakawa and Kawakami, 2008; Kawakami et al., 2010). In fact, the GAL4/UAS system has proven to have great utility for *in vivo* imaging and the manipulation of neural activity in zebrafish (Douglass et al., 2008; Arrenberg et al., 2009; Zhu et al., 2009; Schoonheim et al., 2010; Muto et al., 2011, 2013). However, GAL4/UAS systems also have a disadvantage. It has been reported that the methylation at CpG nucleotides in the UAS sequences could silence the transgene expression in zebrafish that is offspring of the founder (Goll et al., 2009; Akitake et al., 2011; Pang et al., 2015). Although tryptophan repressor (TrpR) and its upstream activation sequence (tUAS) was proposed as an alternative gene expression system to overcome the silencing effect of Gal4/UAS, TrpR/tUAS system gave toxic effect to developing zebrafish may be due to strong transcriptional activity or toxic effect of TrpR protein (Suli et al., 2014). Four distinct Gal4 binding sites that placed in tandem (4x nr UAS) drove high levels of reporter expression with significantly less susceptible to the methylation compare to fourteen tandem copies of UAS (Akitake et al., 2011). Therefore, the fewere non-repetitive UAS sequence could be useful to circumvent the silencing by the methylation.

### The Non-invasive Imaging of Neuronal Excitability

Inhibitory glycinergic and GABAergic synaptic inputs critically regulate neuronal excitability. Electrophysiological techniques are available for the monitoring of single neuron excitability through membrane potential and synaptic current recordings; however, it is difficult to investigate network excitability using this approach. An alternative approach is the monitoring of Ca^2+^ transients as a surrogate of excitability. To this end, genetically encoded calcium indicators have been used for Ca^2+^ imaging in zebrafish (Muto et al., 2011). GCaMP, the most widely used genetically encoded calcium indicator, is a fusion protein of circularly permuted EGFP, calmodulin, and calmodulin-interacting M13 peptide (Baird et al., 1999; Nakai et al., 2001). Although the fluorescence intensity of circularly permuted EGFP is very low, conformational changes induced by Ca^2+^ binding to calmodulin lead to the enhancement of fluorescence intensity and improved detection. The sensitivity of this method has been improved by the generation of GCaMP variants, which are now available for research use (Ohkura et al., 2005; Tallini et al., 2006; Tian et al., 2009; Akerboom et al., 2012; Chen et al., 2013; Muto et al., 2013). Muto et al. (2011) have successfully used this technique to visualize the alternating activation of spinal motor neurons during spontaneous coiling, and neural activity in the optic tectum during prey capture behavior (Muto et al., 2013).

## Zebrafish Motility Defect Mutants

In the Tübingen screen, 166 mutants showing motility defects between 48 and 60 hpf were identified (Granato et al., 1996). Among them, 63 mutants were assumed to be muscle-defective based on the simple observation of actin-myosin organization (birefringence intensity) under polarized light (Felsenfeld et al., 1990). Mutations in dystrophin, laminin, titin, Hsp90 and the cognate co-chaperone Unc45b were identified in this manner (Bassett et al., 2003; Etard et al., 2007; Hall et al., 2007; Steffen et al., 2007; Hawkins et al., 2008; Guyon et al., 2009). The other 103 mutants with normal muscle structure were divided into two groups: locomotion abnormal and reduced motility (Granato et al., 1996), and assumed to have impairments in the nervous system, neuromuscular junction (NMJ), or muscular functional components. Electrophysiological analyses have been useful in delineating the exact cause of each abnormality. For example, electrophysiological recordings from the *accordion* (*acc*) mutant, a locomotion abnormal mutant, revealed normal neuronal outputs and thus implicated deficits in the functional components of the muscle (Hirata et al., 2004). In the *atp2a1* mutant, it was determined that the simultaneous contraction of the bilateral trunk muscles results from the impaired clearance of cytosolic Ca^2+^ by the sarcoplasmic reticulum Ca^2+^-ATPase SERCA1 (Gleason et al., 2004; Hirata et al., 2004). Several other muscle mutants (*relaxed*, *relatively relaxed*) have exhibited defects in excitation-contraction coupling (Schredelseker et al., 2005, 2009; Zhou et al., 2006; Hirata et al., 2007).

Six abnormal locomotive mutants (*bandoneon*, *ziehharmonika*, *bajan*, *diwanka*, *quetschkommode*, and *expander*) were previously compared with the *acc* mutant (Granato et al., 1996). In contrast to *acc*, electrophysiological recordings from the *bandoneon* (*beo*) mutant showed aberrant, arrhythmic fluctuations in response to tactile stimulation, indicating a defect in CNS output (Hirata et al., 2005). As will be described in detail, a molecular genetic study revealed that alteration of the *glrbb* gene is responsible for the *beo* mutant, and thus *beo* exhibits defects in glycinergic synaptic transmission (Hirata et al., 2005). Of note, *beo* is the only known *acc* subgroup mutant with dysfunctional glycinergic transmission (**Table 2**).

**Table 2:** Accordion mutants.

Mutant	Responsible gene	Alleles	Substitutions	Cause of defective behavior	References
*accordion* *acc*	Sarcoplasmic reticulum Ca^2+^ ATPase SERCA1 gene (*atp2a1*) on chromosome 3	dta5 mi25i mi289a tc249a ti284a tm286 tn218b tp72x tq206 ty20	G598V I97N T848I Unknown Unknown Unknown Unknown Unknown S766F Unknown	Impaired clearance of cytosolic Ca^2+^ in muscle cells.	Granato et al., 1996; Gleason et al., 2004; Hirata et al., 2004; Masino and Fetcho, 2005; Olson et al., 2010

*bajan* *baj*	Choline acetyltransferase gene (*chat*) on chromosome 13	tf247	IVS2-2A > C	Attenuated transmission at neuromuscular junction	Granato et al., 1996; Wang et al., 2008

*bandoneon* *beo*	GlyR β subunit gene (*glrbb*) on chromosome 14	tp221 tw38f ta86d ta92 tm115 tf242 tu230 mi106a	Y79X L255R Y79X K343X Q87X Y79D Allele lost R275H	Dysfunction of glycinergic synaptic transmission on spinal motor neurons due to defect in clustering formation of glycine receptor at synapses	Granato et al., 1996; Hirata et al., 2005; Ganser et al., 2013

*diwanka* *diw*	Procollagen lysin 2- oxoglutarate 5-dioxygenase 3 gene (*plod3*) on chromosome 23	ts286 tv205a tz290	Q608X IVS4-2A > G W447X	Defect in primary motoneuron projection. All primary motor axons fail to exit the spinal cord	Granato et al., 1996; Zeller and Granato, 1999; Zeller et al., 2002; Schneider and Granato, 2006

*expander* *exp*	Unknown	tu12	Unknown	Unknown	Granato et al., 1996

*quetschkommode* *que*	Dihydrolipoamide branched chain transacylase E2 (*dbt*) gene on chromosome 22	ti274	IVS6 + 1G > A	Abnormal output from CNS. The abnormality may be result of decrease in neurotransmitter glutamate level within CNS due to dysfunction of amino acid metabolism	Granato et al., 1996; Friedrich et al., 2012

*ziehharmonika* *zim*	Acetylcholine-esterase gene (*ache*) on chromosome 7	sb55 tf222a tm205 tm206	S226N G198R Y139X Allele lost	Reduction of acetylcholine receptor clustering at neuromuscular junction	Granato et al., 1996; Behra et al., 2002; Downes and Granato, 2004

In addition to *beo*, the *shocked* (*sho*) mutant also shows reduced mobility due to a defect in glycinergic synaptic transmission. The *sho* gene encodes the glycine transporter GlyT1 (Cui et al., 2005; Mongeon et al., 2008). Based on its function, the loss-of-function mutation of GlyT1 should increase the availability of extracellular glycine in the CNS. Over-activation of the glycinergic synapse by elevated extracellular glycine can suppress the neural network responsible for motility, and therefore result in a phenotype of reduced mobility. Of note, strychnine, a specific inhibitor of GlyR, partially restores normal neuronal activity in *sho* mutants (Cui et al., 2005). Other mutants with reduced mobility have gene mutations unrelated to glycinergic transmission (**Table 3**).

**Table 3:** Mutants with reduced locomotion.

Mutant	Responsible gene	Alleles	Substitutions	Cause of defective behavior	References
*sofa potato* *sop*	Acetylcholine receptor δ subunit gene on chromosome 24	tj19d ts29 tf207c	L28P Unknown Unknown	Loss of neuromuscular transmission due to defect in acetylcholine receptor clustering	Granato et al., 1996; Ono et al., 2001, 2004

*relaxed* *red*	Dihydropyridine receptor β_1a_ subunit gene (*CACNB1*) on chromosome 3	ts25 mi90	W451X Y283X	Defect in excitation-contraction coupling	Granato et al., 1996; Zhou et al., 2006; Schredelseker et al., 2009

*nicotinic* *receptor* *nic*	Acetylcholine receptor α subunit gene on chromosome 6	tk48d	Unknown	Loss of neuromuscular transmission due to defect in acetylcholine receptor clustering	Granato et al., 1996

*heart attack* *hat*	Unknown	te313	Unknown	Unknown	Granato et al., 1996

*herzschlag* *hel*	Titin gene (*ttna*) on chromosome 9	tg287	Unknown	Unknown	Granato et al., 1996; Myhre et al., 2014

*unplugged* *unp*	Muscle specific receptor tyrosine kinase gene on chromosome 10	te314b	Unknown	Defect in initial outgrowth of motor axons	Granato et al., 1996; Lefebvre et al., 2007; Jing et al., 2009

*shocked* *sho*	Glycine transporter 1 gene (*slc6a9*) on chromosome 2	ta51e te301 ta229g	Unknown C893Y G81D	Over-activation of glycinergic synapse by elevated extracellular glycine level	Granato et al., 1996; Luna et al., 2004; Cui et al., 2005; Mongeon et al., 2008

*twitch once* *two*	Acetylcholine receptor clustering factor rapsyn gene (*rapsn*) on chromosome 18	th26e tm335 tq265b	G130E Unknown Unknown	Loss of neuromuscular transmission due to defect in acetylcholine receptor clustering	Granato et al., 1996; Ono et al., 2002, 2004

*alligator* *ali*	RING finger protein 121 gene (*rnf121*) on chromosome 21	tm342 mi500	L39X V232A	Defect in the excitability of sensory Rohon-Beard neurons	Granato et al., 1996; Ogino et al., 2015

*fakir* *far*	α subunit of voltage-gated calcium channel 2.1b gene (*CACNA1Ab*)on chromosome 11	tm154	L356V	Reduced synaptic transmission between Rohon-Beard neuron and interneuron	Granato et al., 1996; Low et al., 2012

*macho* *mao*	Pigk gene (*pigk*) on chromosome 2	tt261	M1T	Defect in the excitability of sensory Rohon-Beard neurons	Granato et al., 1996; Carmean et al., 2015

*steifftier* *ste*	Unknown	tf220	Unknown	Defect in the excitability of sensory Rohon-Beard neurons	Granato et al., 1996

*crocodile* *cro*	Unknown	tw212d m148 s3556	Unknown Unknown Unknown	Unknown	Granato et al., 1996

*schlaffi* *sla*	Unknown	ty112 th239 tg230	Unknown Unknown Unknown	Unknown	Granato et al., 1996

*slumber* *slm*	Unknown	tt208 tm221 tm132c	Unknown Unknown Unknown	Unknown	Granato et al., 1996

### Defective GlyR Clustering in the *bandoneon* Mutant

The tubingen mutant screen identified seven mutant alleles (tp221 = Y79X, tw38f = L255R, ta86d = Y79X, ta92 = K343X, tm115 = Q87X, tf242 = Y79D, and tu230 = lost) of *glrbb* (Granato et al., 1996; Ganser et al., 2013). In addition, we have isolated an eighth allele (mi106a = R275H) in a previous mutagenesis screen (Hirata et al., 2005). These zebrafish *glrbb* mutants were named *bandoneon* after the South American accordion-like instrument. Phenotypically, the *bandoneon* mutations show simultaneous contraction of both axial muscles instead of alternating in response to touch stimuli; therefore, the mutant body length is shortened by tactile stimuli, similar to the movement of an accordion (Hirata et al., 2005). Since axial muscle contractions are controlled by the reciprocal inhibition of the left and right sides of the spinal cord, inhibitory synaptic transmission is required to produce alternating contractions for swimming (Grillner, 2003; Roberts et al., 2008). As abovementioned, GlyR clustering at synaptic sites is necessary for effective glycinergic transmission and motor pattern generation. In *bandoneon* mutants, GlyR cluster immunostaining in the spinal cord has a diffuse appearance rather than a clustered appearance, suggesting that GlyRb is necessary for the synaptic aggregation of αβ GlyRs in zebrafish (Kirsch et al., 1993; Meyer et al., 1995; Feng et al., 1998; Kim et al., 2006).

### Defective GlyT1 in the *shocked* Mutant

*Shocked* (*sho*) carries a mutation in *slc6a9*, which encodes GlyT1. Three mutant alleles of *slc6a9* were identified in the Tübingen screen (ta229g = G81D, te301 = C305Y and ta51e = unknown), and these *sho* mutants exhibit trunk twitching instead of swimming in response to tactile stimuli at 2 dpf (Granato et al., 1996; Luna et al., 2004; Cui et al., 2005; Mongeon et al., 2008). In addition, the frequency of spontaneous coiling is reduced and the escape contraction at 1 dpf is abolished in *sho* mutants. An electrophysiological analysis revealed that tactile stimuli induces arrhythmic muscle activation in *sho* mutants rather than the rhythmic depolarization observed in wild-type muscle (Cui et al., 2004). The ta229g allele, which produces the strongest phenotype, results from a G81D missense mutation that disrupts GlyT1 function (Cui et al., 2005). Increased extracellular glycine is thought to be the cause of the *sho* mutant phenotype. This presumption is supported by the following observations. First, the perfusion of glycine-free cerebrospinal fluid following the removal of the dorsal roof of the fourth ventricle recovers touch-evoked swimming in *sho* mutants, and this effect is lost when glycine is added to the perfusate (Cui et al., 2005; Mongeon et al., 2008). Second, the application of low concentrations of strychnine to *sho* mutants leads to partial motor recovery, similar to observations in GlyT1 knockout mice (Gomeza et al., 2003a; Cui et al., 2005). Thus, a lack of functional GlyT1 and elevated extracellular glycine are likely to underlie the potentiation of glycinergic transmission in the *sho* phenotype.

The GlyT1 knockout mice exhibited severe motor and respiratory defects and die at birth due to the respiratory defect (Gomeza et al., 2003a), whereas the GlyT1 defective zebrafishes exhibit a motility recovery by 4–5 dpf and survive thereafter (Mongeon et al., 2008). The motility recovery was accompanied by a reduction in GlyR expression that leads to a decrease in the amplitude of inhibitory potentials enhanced by the GlyT1 mutant (Mongeon et al., 2008). Although the alteration of GlyR expression may represent compensatory mechanisms, the molecular basis for this compensation is not revealed.

Glycinergic transporter 1 dysfunction has been posited in the pathogenesis of glycine encephalopathy, which is characterized by respiratory impairment (Gomeza et al., 2003a; Applegarth and Toone, 2004, 2006; Harvey et al., 2008b), and schizophrenia which may be arose from hypofunction of NMDAR (Krystal et al., 1994; Lahti et al., 2001; Tsai et al., 2004; Dalmau et al., 2007). Animal models are clearly required to investigate the biological roles of GlyT1 in the context of human disorders, and zebrafish may provide particular advantages in future efforts. For example, while GlyT1 knockout mice die on the first postnatal day due to severe respiratory deficits (Gomeza et al., 2003a; Tsai et al., 2004), zebrafish *sho* mutants can survive well into adulthood through careful feeding and are thus useful as a physiologically relevant animal model for GlyT1 dysfunction.

## Conclusion

Functional GlyRs are α homomeric or αβ heteromeric pentamers. Electrophysiological studies of α subunits in cultured cells have shown the difference in conductance and kinetics of each α subunit (Takahashi et al., 1992; Bormann et al., 1993; Rajendra et al., 1995; Beato et al., 2002; Mangin et al., 2003; Burzomato et al., 2004; Zhang et al., 2015). Moreover, both in zebrafish and in mammals, each α subunit exhibits a distinct pattern of temporal and spatial expression in the CNS. This diversity of the α subunits suggests that GlyRs play various roles in physiological functions. However, the details of these physiological roles are not well understood. Several studies have identified GlyR subunit selective modulator in Cannabinoids and endocannabinoid (Hejazi et al., 2006; Yang et al., 2008; Xiong et al., 2012), in compounds isolated from ginkgo and Australian marine sponges (Balansa et al., 2010, 2013a,b; Maleeva et al., 2015). Furthermore, recent publications have reported that heptapeptide exhibit the subunit selective modulating effects on α1β and α3β heteromeric receptor with zinc ion dependence (Cornelison et al., 2016). These subunit-selective modulators would be useful tool to investigate the physiological roles of GlyR subunits in CNS. Together with the selective modulator, losses of function mutants of each GlyR subunit are powerful tools for uncovering the specific functions of each GlyR subunit. Now, zebrafish provide a cheaper, more convenient and highly useful alternative animal model for the study of GlyRs and glycinergic transmission. So far, the *glrbb* and *slc6a9* mutants have been used to investigate the glycinergic synapse. Future work can further utilize CRISPR/Cas9-mediated gene disruption in zebrafish. In addition, *in vivo* observations of the glycinergic synapse in living zebrafish are a powerful method for the study of glycinergic synapse formation and plasticity. Taken together, a combination of readily available genetic mutants and innovative imaging techniques in zebrafish is sure to accelerate our understanding of glycinergic neurotransmission in the future.

## Author Contributions

All authors listed, have made substantial, direct and intellectual contribution to the work, and approved it for publication.

## Conflict of Interest Statement

The authors declare that the research was conducted in the absence of any commercial or financial relationships that could be construed as a potential conflict of interest.
